# Exploring the Experience of Student Researchers and Family Partners Working as an Interdisciplinary Team

**DOI:** 10.1177/23779608241274222

**Published:** 2024-08-26

**Authors:** Karsten Berg, Mariana dos Santos Ribeiro, Hadiya Huijer, Sandi Whitford, Schroder Sattar, Roslyn M. Compton

**Affiliations:** 1 College of Nursing, 7235 University of Saskatchewan, Saskatoon, Saskatchewan, Canada; 2 Faculty of Health Sciences, 12363 Queens University, Kingston, Ontario, Canada; 3 Health Education and Engineering, 5333 University of the Sunshine Coast Faculty of Science, Sippy Downs, Queensland, Australia

**Keywords:** undergraduate, graduate, patient-oriented research, thematic analysis, interdisciplinary

## Abstract

**Introduction:**

Many institutions seek to engage postsecondary students in research to grow future researchers. Despite this common goal, the means to achieve that end is often unknown creating difficulties for students as they seek out research opportunities.

**Objective:**

This article will share first-hand reports on the experiences of graduate students, undergraduate students, and family partners following their engagement as an interdisciplinary research team.

**Methods:**

This is a qualitative study using individual open-ended interviews with undergraduate students, graduate students, and patient family partners. The research was conducted using a Patient-Oriented Research approach. The transcripts were analyzed inductively by using Braun and Clarke's six phases of reflexive thematic analysis.

**Results:**

Two themes were developed from the analysis. The theme of *Benefits & Facilitators* was developed based on the many positive thoughts, feelings, resiliency, and learning expressed by the students and family partners. The undergraduate students found the experience of “doing” research fulfilling and a great tool for learning how to better use research in practice. Within the second theme *Perceived Hierarchy*, undergraduate students described themselves as helpers, not responsible, and they did not have the power to make decisions.

**Conclusion:**

Given the challenges identified in this study, future efforts in this approach should carefully consider the culture and how to best engage graduate students, undergraduate students, and family partners in research teams.

## Introduction

As knowledge grows, the need for research increases. Undergraduate students are often an untapped resource that could be utilized to support, develop, and perform research at many levels. This represents opportunity to bring undergraduates into research as key members to influence change. Studies on how undergraduates can be engaged in research have been performed, and their findings identify a need for student involvement and mentorship throughout the process (Aponte et al., 2015; Hickey et al., 2019; Vessey & DeMarco, 2008). Undergraduate research is difficult due to students’ interest in learning contrasted with their limitations to learning and performing research independently (Lev et al., 2010; Sadler & McKinney, 2010). This highlights the need for skilled mentors who can provide teaching to guide this process of learning and achieving competence (Petrella & Jung, 2008). Mentors may experience tension when mentees require their presence and availability as challenges occur (Corsby et al., 2022; Murdoch-Eaton et al., 2010). This creates learning experiences for graduates and undergraduates as they negotiate new relationships.

## Literature Review

When working with teams of undergraduate student researchers, there is a natural shifting between the experienced and inexperienced. This creates an environment where the experienced take the lead, providing guidance based on their past knowledge and experience. The dynamics and culture established at the origin of this relationship between mentee(s) and mentor may be critical in addressing expectations concerning the research process (Aponte et al., 2015). Exploration of these dynamics and this culture will provide valuable insight into how graduate and undergraduate students view research (Ayoola et al., 2017).

By collecting insights from graduate and undergraduate students and examining their experiences, researchers can better understand the obstacles that impact students’ interest in pursuing and engaging in research activities (Loke et al., 2014). In this way, the complex relationship between students involved in research and the research process itself needs to be considered and addressed to encourage undergraduate students to look for and accept research opportunities (Wells & Cagle, 2009). Individuals with lived experience should be considered in this process as they have first-hand knowledge of the area of research and thus are critical collaborators, producers, and managers of knowledge (Hemming et al., 2021).

This project studied the benefits of bringing undergraduate students together with graduate students to create teachable moments. It was hypothesized that by engaging students in research, they would experience the value of research and begin to incorporate these new insights into their emerging practice. By giving undergraduate students research opportunities, it was thought they would become more informed and involved users of research and would be increasingly involved in emerging practices (Imafuku et al., 2015). A recent search of the literature reveals a lack of research examining how health sciences undergraduate students can benefit from doing research before graduation. This lack of familiarity with research creates a lack of interest or drive for undergraduate students to engage in and perform research (Mellor et al., 2013). For undergraduate students, research may be seen as something to do to advance their career or gain higher education (Murdoch-Eaton et al., 2010).

Undergraduate research is seen as a benefit to students increasing their postgraduate research productivity and contributions to their related fields (Murdoch-Eaton et al., 2010). Despite potential benefits of undergraduate research, and how they can be supported as they learn to conduct research for the first time. Much of what researchers understand about this topic is derived from fields such as education, business, and engineering. Research generated from these realms often have a vastly different focus than healthcare and thus data generated from these studies is harder to interpret in the healthcare environment (Berri et al., 2012; Dooley et al., 2004; Gilmore et al., 2015; Hathaway et al., 2002; Horowitz & Christopher, 2013; Klein et al., 2008; Shostak et al., 2010; Stamp et al., 2015; Wylie et al., 2019). Kaul et al.'s (2016) study indicated that students could lose motivation and interest in research if unsupported in complex projects. Adebisi (2022) reviewed 15 studies on student research engagement and found the most common barriers were lack of knowledge and skills, minimal faculty support, mentorship, and motivation to participate in research as an undergraduate. The authors found that overcrowded student schedules, and frequent scarcity of programs which would accept undergraduate researchers were other factors.

Murdoch-Eaton et al. (2010) performed a study that looked at engagement within 905 research projects in the United Kingdom. The authors looked at development of four main skills (research methods, information gathering, critical analysis, and review and data processing) to measure student engagement and found 52% of projects engaged one or more skills, while only 13% engaged all four skills. This result indicates that most students were given an unbalanced view of research, one which does not give students a true idea of what research is. Murdoch-Eaton et al. (2010) also conducted focus groups for participating students and found many students developed a negative view of research following their experience. One student described their research experience as akin to providing “free labour” (p. 157). The study findings concluded that students should be included in multiple parts of the research process and their contributions should not be limited to monotonous data collection to encourage continuing engagement of students in research.

A literature review performed by Sadler and McKinney (2010) focusing on undergraduate students found students who were fully engaged in research throughout the research process reported positive perceptions of research as opposed to their less-involved peers. However, despite these positive changes, there was significant variability based on the quality of mentorship received (Sadler & McKinney, 2010). This need is echoed by Petrella and Jung (2008) who noted the importance of mentorship when introducing undergraduate students to research. This was noted to be important for undergraduate researchers to help them develop a deeper understanding of the research process. This highlights the need to not only engage students in research but also to mentor and support them throughout the process.

Family partners (commonly known as patient partners, in patient-oriented research) are experts by lived experience. Family partners are a type of informal carer who provide care to family members, friends, and neighbors (Rocard & Llena-Nozal, 2022). Their acquired skills and experience are often missing among researchers, who instead bring analytical skills and a research perspective. The partnership between these two parties brings together clinical skills, with a personal perspective on where research could provide the most impact (Hewlett et al., 2006; Tomlinson et al., 2019). Through this close relationship, researchers can ensure they understand the family partner's experience and perform research in areas of priority (Hewlett et al., 2006). The term partner is both intentional and critical to research, as family partners are not intended to just advise but also to participate in all stages of the research process, from applying for grants to review for final publication. It is critical to consider this valuable population and their perspectives in research moving forward (Saskatchewan Center for Patient-Oriented Research, 2019).

The current state of knowledge suggests this research is novel. The authors performed a literature review and found limited research that attempts to understand the perspective of students and family partners working within the area of geriatric care. This research offers value as it examines an area of research seldom explored and provides a new perspective that could inspire further research and development in this area.

## Methods

This study is embedded within a larger study, which was aimed at creating connections between healthcare workers, families, and informal carers. The objective of the original study was to maximize the quality of life and quality of care experienced by older adults, close ones, and staff through informational social media posts. Facebook and Instagram users were the population group targeted for study inclusion by the researchers. The posts addressed topics pertinent to nurses caring with older adults. These topics included mental health, oral health, physical exercise, and nutrition, among others which were identified as relevant by family partners and healthcare professionals. The research team included students from nursing, psychology, cellular physiology, pharmacology, gerontology, and education at various points in their undergraduate and graduate education, as well as family partners. Family partners were not only included for their lived experience but also brought professional experience to the team from their backgrounds of business, education, and horticulture. Yeager (2005) defines interdisciplinary care as multiple disciplines working together to deliver the highest quality of care. Considering this definition, all team members, be they students or family partners, contribute to achieving this goal of the highest quality of care. The team in the original study achieved their goal of releasing a social media post once weekly for 30 weeks and concluded with their final weekly post on August 1, 2023. At the conclusion of the study, there were 50 and 55 followers on Instagram and Facebook pages, respectively.

### Design

This is a qualitative study using individual open-ended interviews with undergraduate students, graduate students, and family partners. Interviews were performed using an interview guide. Using previous work by Burgess et al. (2020) and University of British Columbia Faculty of Medicine (2021), the interview guide was cowritten by the research team. The interview guide questions were designed to encourage interviewees to openly share their experiences as a member of the research team. The research was conducted using a Patient-Oriented Research approach. This involved students and family partners throughout the research process. The research was conducted in a way that was respectful of, and responsive to the needs of individual students, and the family partners guided research decisions (Seely & Grinspoon, 2017). Based on this approach, the authors adopted the use of researcher as participant as the guiding principle for the research (Probst, 2016). To ensure trustworthiness, all interviews were conducted by a single interviewer employing a semistructured interview guide. This approach facilitated the participants’ ability to articulate their experiences freely. The consistency in the interview process preserved the integrity of the data, as the interviewer refrained from altering questions in subsequent interviews to avoid introducing bias or selectively emphasizing particular themes (Elo et al., 2014). Using this process, the authors hoped to provide context to the experience under study.

### Research Question

What are the experiences of graduate and undergraduate learners and family partners conducting research in an interdisciplinary patient-oriented research team?

### Sample

The research entailed collecting data from an established team working collaboratively to achieve a research goal. The team under study was an interdisciplinary, culturally diverse team of five health sciences students, one education student, and two family partners. Each team member was invited to participate in an individual interview. The team had extensive experience working together as they met weekly for one hour over Zoom over a period of 30 weeks. The team committed to setting aside additional time for review, reflection, and revision of posts during the week. This process was completed through a shared WhatsApp group, email, and collaboratively over Canva. Although the students and family partners created posts together, each member was assigned a range of roles including marketing, design, meeting facilitation, and topic area research.

### Inclusion Criteria

All members of the interdisciplinary research team were invited to participate. No one was excluded from participating in this study.

### Data Collection Setting

The research was performed in Canada, and data were collected between June and August of 2023 by the first author who was not a member of the research team being interviewed. Due to the interprovincial nature of the team, all data collection occurred digitally using the videoconferencing platform (Zoom). This allowed the interviewer to engage each member of the team where they lived, worked, and practiced. Although the nature of the discussion was not particularly distressing, the students and family partners were sharing their experiences, and this was respected throughout the interview process.

### Data Analysis

The interviews were recorded and transcribed verbatim by the first author. Transcripts were checked against the original audio recordings to ensure accuracy and were sent for verification to students and family partners to ensure their validity. The transcripts were analyzed inductively by K.B., M.S.R., H.H., and S.W. (the analysis team), using Braun and Clarke's (2021) six phases of reflexive thematic analysis. This reflexive approach fits well with the Patient-Oriented Research approach as both ideals shift the focus toward what the individual is saying (Rao, 2022). This contrasts with more traditional approaches where the researcher collects data and performs the analysis in isolation from the population being studied (Hemming et al., 2021). By being engaged as participants, the students and family partners were enabled to be researchers.

Phase 1 analysis was completed by the analysis team. In this phase, the analysis team became familiar with the data by reading and rereading the transcripts. The analysis team was provided with one deidentified transcript to independently analyze and produce notes. Notes were encouraged in any form and were to be open and include reflections. Once the analysis team had reviewed the interview transcript, the notes and reflections were discussed as outlined by Braun and Clarke (2021). This added substantial depth to the review of the data as each person brought multiple perspectives and experiences. This depth of perception leads in many cases to a richer understanding of the content.

The discussion by the analysis team provided insight into the data. An interesting insight was how students and family partners related to the experiences described, quoting passages from the data that resonated with their experiences. The discussion became particularly rich when the analysis team had contrasting thoughts and interpretations of the data. Notes were combined and collated using an online Padlet board to facilitate coding.

At the beginning of coding, the analysis team reviewed the coding process as described by Braun and Clarke (2021). Boyatzis (1998) describes a code as “the most basic segment, or element, of the raw data or information that can be assessed in a meaningful way regarding the phenomenon” (p. 63). Coding started with discussion of potential codes by making connections and organizing notes from the online Padlet board. As codes emerged, they were noted, discussed, and either abandoned, revised, or combined, as advised by Braun and Clarke (2021). The final codes were developed and were found to be supported by an original code of the analysis team. The coding was checked and confirmed by R.M.C and S.S. Examples of the refining of codes can be found in Table 1.

**Table 1.** Final Codes.

Final code	Author 1 original code	Author 2 original code	Author 3 original code
Unclear Roles	Perception of shifting roles	Team member roles absent at inception	Lack of role definition at the onset of the study
Shifting Leadership based on Hierarchy	Shifting leadership	Perception of team leadership and ownership of project inconsistent with actual intended team leadership	Team leadership changes based on individuals present at the meeting
Team Conflict	Distress when the project does not go as planned	Lack of role clarity led to conflict	Workflow and conflict due to undefined rules
Hazy Decision Making	Inconsistency in team decision-making	The perception that team decisions needed to be approved by the “supervisor"	Does the team make decisions or the team leader?
“Just a Helper”	Unclear roles, a “helper”	Lack of role clarity regarding self as a researcher	View of self as a helper, not as a researcher
Lack of Undergraduate Ownership	Can't have ownership as is helper, not a researcher	Sense of responsibility to create a post but no ownership of the post	Identifies self as responsible but a graduate student as having ownership
Competing Priorities	Identified lack of initiative from team members	Project “was no one's top priority"	Difficulty in engaging family partners based on student schedule
Disorganization	Disorganization impacting the project	Early disorganization led to unequal workflow	Project attendance was sporadic which led to disorganization
Communication Concerns	Fear of graduate students dominating the conversation	Undergraduates did not readily engage and needed to be coaxed to speak	Undergraduates not being willing to speak up hindered productivity
Fluctuating Attendance	Difficulty in engaging family partners based on student schedule	Challenges arose from inconsistent attendance and lack of organization	Family partner did not feel it was important to attend consistently due to the availability of the team
Leadership role unwanted/burdensome	Leadership can be a tiresome position	Being the sole leader was unwanted by graduate student	No one would take charge if leaders were not present. Student response was just to wait until leaders arrived
Undergraduate loss of focus toward research objective and purpose.	Confusion regarding the goals of the research	Undergraduate team members’ perspectives distanced from research goals	Undergraduates lack understanding of plan to achieve goals
Unequal workflow	Inconsistency in team members	Team members contribute to posts in varying degrees	Teamwork is unproductive due to a lack of individual contributions outside of meeting times

Once consensus was reached, the analysis team began theming the data. The analysis team continued to organize, group, and make connections within the codes in the online Padlet board. This follows Braun and Clarke's (2021) recommendation that codes should be organized visually into groups with connections drawn between different ideas. The data were clustered when strong relationships were apparent. These formed initial themes, which were carefully examined, revised, or abandoned, as part of the reflexive thematic analysis process (Braun & Clarke, 2021).

Once a set of themes are developed, Braun and Clarke (2021) recommend another review of the data. This step validated themes against the experience of the students and family partners described in the data. Following this review, themes were accepted and agreed upon by the analysis team. The themes were checked and confirmed by R.M.C and S.S.

### Institutional Review Board Approval

Ethical approval for the study was obtained from the Behavioural Research Ethics Board (BEH#3646) at the University of Saskatchewan. Interviews were conducted with utmost attention to ethics, and with a high degree of sensitivity.

## Results

### Sample Characteristics

The sample included a single interdisciplinary research team of which 100% participation was attained. The sample consisted of seven females and one male. The participants were at various levels of their studies, including 3^rd^ and 4^th^ year of undergraduate programs, graduate students in their final year of a master's program, a third-year Ph.D. student, and experts by lived experience. The study hosted students and family partners from various cities within the provinces of Saskatchewan and Ontario.

### Research Question Results

Two themes were developed from the analysis, *Benefits & Facilitators*, and *Perceived Hierarchy*, as well as one emerging theme, *Different View of Research*.

### Benefits & Facilitators

The theme of *Benefits & Facilitators* was developed based on many positive thoughts and feelings that were expressed by students and family partners which showed their resiliency and learning. This theme highlights how many positive and developmental experiences were generated for this team of student researchers. The experience of developing research from start to finish was often mentioned as one of the keystone benefits of the project for the students. This was aptly highlighted by one undergraduate student who stated. “At first, I didn't know much about conducting research. I have always shown up somewhere in the middle. Being part of it from the start, I now know how much of a hassle it is to get through ethics, writing a grant, and writing a proposal. Then having them respond, having to change little things, and having to follow the protocols from start to finish. This gives me more of a feeling of how our research is supposed to go… It's an eye-opening experience. It is not like anything you learned at school.”

Many undergraduate students described performing research as fulfilling and as a great tool for learning how to use research in practice. “I can follow a research paper much faster now, ever since I started working with the group. You get appreciative of things people do when they research once you have done it yourself. You understand why they're doing this.”

The undergraduate students expressed satisfaction working interdisciplinary with diverse individuals. “I felt in an interprofessional team, I got to think about topics holistically and look at them from different aspects. Not just, how do we complete the study and end up with the best results? But what does this mean outside of the study sphere? Or what does this mean for people in real life? Getting to see that real-world application was nice.”

Undergraduate students expressed satisfaction with contributions of family partners. “Having a family partner on our team has been a great help because we're discussing different things like, how can teams communicate better with patients? The family partner will talk about how her family was in long-term care. How did that look for them? I think the family partners provide insight into what it's like for the patient.”

The project was an excellent means for encouraging undergraduate students to conduct research and to value research postgraduation. “I did not think I was interested in research, before I began. I didn't know at all what research meant and when I started. It has made me open to the possibility of pursuing research in the future.”

The students developed a connection with the work and with each other. This made them want to continue working on this project. “I started with this team as an intern for two months. I ended up continuing because I loved the team dynamics. I loved hearing different experiences every Friday morning and was so excited for the Friday morning meetings.”

Undergraduate students spoke positively about the mentorship received by graduate students. “It's different. Ph. D. and graduate students are knowledgeable, and they guide me. They help me see how the world works. As an undergraduate, I don't know how to be a researcher or how a research proposal works.” The students also mentioned the importance of mentorship they received from family partners. “I realized family partners, individual family members, and caregivers have lots of experience and personal stories to share that we can benefit from. Sometimes it goes overlooked because they don't have a medical designation, but they have lots of experience and knowledge to share.”

Undergraduate students expressed their research could have a positive influence and help others. It was emphasized that positive change was the most important outcome of the project. “Creating posts for an audience and having them read the post and feel less anxious and overwhelmed. It makes me happy and makes me want to do more posts.”

For most students, this was their first opportunity to work as a member of an interdisciplinary team and they expressed great appreciation for this experience. “I feel I bring a different aspect to group meetings since we are working towards the same goal. We bring different aspects. It is nice to see different points of view.”

### Perceived Hierarchy

Undergraduate students described themselves as helpers, not responsible, and that they did not have power to make decisions. The hierarchy is perceived rather than actual, as the opposite was meant to be true. The project was created with the intention that undergraduate students would lead the project and make all decisions. The graduate students and family partners were meant to act as mentors.

When asked about their role in the team nearly all the undergraduate students described themselves as “just a helper.” This perceived subservient helper role was an interesting finding, as the undergraduate students were far from helpers. They were core members of the team, as it was undergraduate students who were tasked with developing, formatting, and pitching posts to the graduate students and family partners each week. This was found to be frustrating for graduate students and family partners as the goal of the project was for it to be undergraduate student led. The following statements by undergraduate students express this difference in views. “I think my role is just to contribute any ideas I think are appropriate.”; “To be honest, I am not sure of my exact role, besides helping.” The preceding quote illustrates how undergraduate students were unable to adopt roles. The quote was recorded 26 weeks into the 30-week project.

The idea of “just a helper” was driven home by an undergraduate student. “I think this project is [graduate student]'s project. Right? I think [graduate student] is the one who makes the final decisions because this project isn't spearheaded by me.” The idea that graduate students were in control was repeated throughout the interviews by the undergraduate students. Students described the team structure as open, inclusive, and collaborative. However, when asked about the final product, and how final decisions were made, undergraduate students indicated all final decisions were made by the graduate student who was perceived to be the leader. “Everyone is working on consensus, but the leader would try to make everyone satisfied. The leader is generally the one who's sharing the post so they would be trying to figure out how they can change the post around.”

This idea was also present among family partners. The team was collaborative, and decision-making was made as a team; however, some members did have ultimate authority. “Well, we tried. The majority usually ruled for sure. But we looked up to the seniors. [They] kind of had veto power.” This idea of perceived hierarchy was consistent across the family partners within the team. “There was always one person who had to be the leader and had to get the ball rolling. [Graduate student] and [graduate student] were the most important part to get our ideas flowing and generated.”

This hierarchical view helped explain the idea of undergraduate students being helpers, under the graduate students. This explains the hesitancy of undergraduate students to take any leadership role. One undergraduate student expressed the following. “I think we all follow [Graduate Student] because we all see [Graduate Student] as a leader. Sometimes we change things, so it suits [Graduate Student]. Honestly, conflict isn't the right word, maybe just agree to disagree.”

Despite this perception that graduate students were the leaders, and perceived to be in a position of power, the graduate students expressed they did not want to lead. “I can see myself as the leader, but it's not something I wanted.” In one case, a family partner shared an experience where she was made the leader for one week. “It was interesting, because [graduate student] wasn't there and [graduate student] wasn’t there, and there wasn't anybody else that was going to be the leader.” They said, “Will, you lead.”

This hierarchical idea fed into team organization. Undergraduate students were slow to or did not adopt negotiated roles within the team, and these roles were hazy. Graduate students identified this conflict and lack of productivity within the team in their interviews. “At the beginning, we had not defined specific roles and we did not know how this is going to work. It was a little bit disorganized, and I remember a few team members felt they had been contributing more than others, and that needed to be addressed.”

These ideas speak to a lack of ownership and commitment to the project by undergraduate students. This was noted by students and family partners as attendance was discussed. One family partner made the following statement. “I want to say they were open and inclusive when we all got together, but things were sporadic, it could have been more organized, and more well attended. When we did all come together, it worked.”

One graduate student expressed frustration with this lack of commitment and compared it with previous projects they had attended. “During a previous project I worked on, we had a clear timeline right from the start. We knew what needed to get done each week and who was going to do it. Everyone stuck to that, and if someone needed help on something because of school or work commitments, other people would step in and do things to help. Whereas this project never exactly felt like that.”

#### Different View of Research

An emerging theme of this research was how individuals can view research differently after engaging in and being exposed to research. This project exposed students and family partners to many facets of research, some of which they had not previously considered “research.” By engaging in this project, both students and family partners expanded their understanding of what research is. This is highlighted by a statement by one of the family partners. “If this is research, then it has changed my idea of what research is and what it looks like. If this is classified as research then I like it a lot, and it opens up new doors.” The undergraduate students expressed this idea of a different view of research. “I do view research differently. I used to see it as something adding to the literature and expanding that database of literature. Now I see it as something where you work on an existing problem and change systems that are in place. I view it as more of an act of doing than I did before.”; “I would say it's both different and better, possibly better, because it is different.”

## Discussion

Through this study, the authors have gained unique insights into the dynamics of an interdisciplinary research team by examining the experiences of graduate and undergraduate students, as well as family partners, engaged in patient-oriented research. One such perspective would be the absence of negative statements from undergraduate students. This absence could be an extension of *Perceived Hierarchy*, which may in part have influenced responses received by the graduate student interviewer. This may explain why undergraduate students’ statements were more positive with less discussion of conflict or issues as compared to graduate students and family partners. This is supported by the finding of Kaul et al.'s (2016) where undergraduate student responses may be unintentionally positive due to the expectation that research brings positive results. This is shown by the data where the graduate student stated “I didn’t feel like everyone was contributing” which shows a sense of conflict.

The undergraduate students all avoided any mention of conflict. One undergraduate student stated “Honestly in conflict isn't the right word. Maybe just agree to disagree.” In their work with undergraduate and graduate students working in teams, Zolfagharian et al. (2011) reported similar issues with implementation and group formation. Zolfagharian et al. recommend that teams of undergraduate and graduate students create their own protocol rather than contributing to an existing one. They recommended that undergraduate and graduate students be highly trained, including hands-on training, required readings, and timely planning to prevent potential issues during the research process. These findings highlight how deeply rooted perceived hierarchy may be.

Based on the original project design, undergraduate students were intended to be the main decision-makers, organizers, and managers of the project. This expectation was communicated with undergraduate students at the outset. The lack of ownership and lack of commitment described by graduate students and family partners may be influenced by undergraduate students’ Perceived Hierarchy. If undergraduate students saw themselves as “just a helper,” they would likely place less emphasis on their participation and be less likely to adopt a role. Hathaway et al. (2002) found undergraduate students were less likely to engage with research when the topic was not one of interest or when mentorship and support systems were lacking.

Hinojosa et al. (2014) examined leader and follower dynamics and found three common types of teams. Hinojosa et al. named these secure teams, insecure-ambivalent teams, and insecure-avoidant teams. Secure teams are represented by close relationships where leaders provide knowledge and promote confidence of team members which results in success as a team. Insecure-ambivalent teams tend to over-rely on their leader appearing dependent on the leader's presence for the team to function. On the other hand, insecure-avoidant teams tend to do their work in isolation from each other, avoiding contact with other team members. Moving forward, teams need to be mindful of individuals coming from diverse perspectives which may complicate this leader–follower dynamic. Teams need to consider any form of hierarchy which imposes a feeling of inferiority on students.

Further, when teams are approaching an arrangement with multiple individuals, they should anticipate potential pitfalls in team dynamics (Hinojosa et al., 2014). Teams that fail to address this may experience ill-defined roles where individuals are unlikely to communicate or have the confidence and ability to complete assigned tasks. Failure to consider these factors may lead to a team that lacks communication and organization and may result in a hierarchical system where some team members experience a diminished sense of collaborative decision-making.

A finding of particular interest was when a family partner was asked “Will you lead” by an undergraduate student. The Saskatchewan Center for Patient-Oriented Research [SCPOR] (2019) has created a measurement tool called Patient-Oriented Research Level of Engagement Tool (PORLET). The tool is used to determine level of engagement of family partners within patient-oriented research teams, where one is inform, and five is empowered. If this tool were to be used with the students it is probable that the family partners in this study would score highly on the PORLET (SCPOR, 2019). This contributes to the theme of Perceived Hierarchy as undergraduate students respect family partners enough to ask them to lead. Alternatively, this may have been attributed to perceived hierarchy, in which students viewed family partners as having more authority, knowledge, and/or experience. Further research is warranted to understand the role of the family partner in student-led patient-oriented research.

Findings from this study highlight the importance of mentorship and the development of rapport. Corsby et al. (2022) describe the challenges of mentorship when students are mentored by other students. A key feature present in student mentorship is the level of education and the development of rapport. The most critical aspect of student mentorship is development of this working relationship and the continuous presence of mentors (Corsby et al., 2022). The results of this study align with those of Corsby et al. (2022), except that this study did not actively investigate student and family partner perceptions of the rapport among team members. The team needed the mentors to be ever present to help them remain on task and guide them through decision-making and assignment of duties. This highlights the need for quality mentorship, which is not only present but also intentional and focused on individual learner needs.

## Strengths and Limitations

To the best of the authors’ knowledge, this is the first study to explore experiences of graduates, undergraduates, and family partners in an interdisciplinary patient-oriented research team. The findings represent an important foundation for further inquiry for research in this area. A strength was the undergraduate students, graduate students, and family partners were interviewed individually. This aided in gathering multiple perspectives. A benefit of taking this approach was each person could speak without being influenced by others. This was important as interviewees may have been unwilling to speak if members of the perceived hierarchy were present.

Another strength of the research was the multiculturalism of the team. The team was mindful of including diverse perspectives irrespective of culture. This may have been a limitation because the interview guide did not include questions concerning culture or cultural understandings of team membership and leadership. This omission was noted during the analysis and discussion when *Perceived Hierarchy* was identified as a theme and warrants further research.

A significant limitation of this paper is it did not consider teams as multicultural. This is especially important to research in healthcare due to the multicultural nature of healthcare teams. Culture should be carefully investigated and considered when perspectives around teamwork and team functioning are considered. Shin et al. (2016) examined this idea of culture matters and found team performance hinges on support and organizational processes. Shin and colleagues (2016) emphasize that teams that promote culture often tend to be more innovative, flexible, and more willing to embrace new ways of working. Embracing culture allows teams to adapt to complex problems and changes in the work environment (Shin et al., 2016). Considering the increased complexity of managing multicultural teams, leaders need to recognize the challenges of working within these teams and develop support to ensure the success of the team.

Another potential limitation was that the interviewer was a graduate student. If the interviewer was considered part of the perceived hierarchy, then undergraduate students may have modulated their responses to fit what they thought the interviewer wanted to hear. This may have made undergraduate students less likely to disclose negative thoughts and feelings about the project and focus primarily on positive aspects (Setiadi et al., 2017).

## Implications for Practice

The research indicated there are many benefits and facilitators to including undergraduates and graduate students in research. This inclusion benefits students, allowing them hands-on training performing research, and a better understanding of each facet of the research process. This learning follows recommendations for the application of evidence to practice (Loke et al., 2014). Performing research helps students become better consumers of research and aids their understanding and application of this research (Devido et al., 2020). These findings are consistent with the stated experience of students in this study.

Despite these positive changes, students who do not engage in research are less likely to engage in evidence-based practice after graduation (Lehane et al., 2019). Lehane et al. (2019) explain that the difficulty in teaching these practices lies in development and organization of content, approach to education, and availability of quality teachers and mentors. These factors are both available and easily accessible within educational institutions which make institutions well-suited to teaching research skills (Loke et al., 2014). This indicates evidence-based practice is best taught through research which is supported by mentors at educational institutions.

## Conclusion

Engaging graduate students, undergraduate students, and family partners in research positively influences their learning, understanding, and engagement with research and may promote undergraduate students’ interest in performing and engaging in research after graduation. Students and family partners in this study reported a new and often different understanding of what research is, and how it can be used to improve the patient care environment. Engaging students in research is an important strategy to integrate evidence into practice. Individuals following this approach should carefully consider culture and how to best embrace culture within their team. This study adds to the body of knowledge for including graduate, undergraduate, and family partners in research teams.
